# Assessment of expected breeding values for fertility traits of Murrah buffaloes under subtropical climate

**DOI:** 10.14202/vetworld.2015.320-325

**Published:** 2015-03-12

**Authors:** Soumya Dash, A. K. Chakravarty, Avtar Singh, Pushp Raj Shivahre, Arpan Upadhyay, Vaishali Sah, K. Mahesh Singh

**Affiliations:** 1 Dairy Cattle Breeding Division, National Dairy Research Institute, Karnal, Haryana, India; 2 Division of Animal Genetics, Indian Veterinary Research Institute, Izatnagar, Bareilly, Uttar Pradesh, India

**Keywords:** critical heat stress zone, expected breeding value, murrah buffaloes, temperature humidity index

## Abstract

**Aim:**

The aim of the present study was to assess the influence of temperature and humidity prevalent under subtropical climate on the breeding values for fertility traits *viz*. service period (SP), pregnancy rate (PR) and conception rate (CR) of Murrah buffaloes in National Dairy Research Institute (NDRI) herd.

**Materials and Methods:**

Fertility data on 1379 records of 581 Murrah buffaloes spread over four lactations and climatic parameters *viz*. dry bulb temperature and relative humidity (RH) spanned over 20 years (1993-2012) were collected from NDRI and Central Soil and Salinity Research Institute, Karnal, India. Monthly average temperature humidity index (THI) values were estimated. Threshold THI value affecting fertility traits was identified by fixed least-squares model analysis. Three zones of non-heat stress, heat stress and critical heat stress zones were developed in a year. The genetic parameters heritability (h^2^) and repeatability (r) of each fertility trait were estimated. Genetic evaluation of Murrah buffaloes was performed in each zone with respect to their expected breeding values (EBV) for fertility traits.

**Results:**

Effect of THI was found significant (p<0.001) on all fertility traits with threshold THI value identified as 75. Based on THI values, a year was classified into three zones: Non heat stress zone(THI 56.71-73.21), HSZ (THI 75.39-81.60) and critical HSZ (THI 80.27-81.60). The EBVfor SP, PR, CR were estimated as 138.57 days, 0.362 and 69.02% in non-HSZ while in HSZ EBV were found as 139.62 days, 0.358 and 68.81%, respectively. EBV for SP was increased to 140.92 days and for PR and CR, it was declined to 0.357 and 68.71% in critical HSZ.

**Conclusion:**

The negative effect of THI was observed on EBV of fertility traits under the non-HSZ and critical HSZ Thus, the influence of THI should be adjusted before estimating the breeding values for fertility traits in Murrah buffaloes.

## Introduction

India is the largest producer of milk in the world and buffaloes contribute the highest (56.64%) share to milk production [1]. According to 19^th^ livestock census [2], the population of buffaloes are quite uprising, and there are 108.7 million numbers of buffaloes present in India. Buffaloes are considered as a triple purpose species producing milk, meat and draft power for agriculture work. One of the most limiting factors in dairy production in the subtropical climate is heat stress. Heat stress is defined as the combination of environmental parameters producing conditions that are higher than the temperature range of animal’s thermal neutral zone [3]. Buffaloes are highly susceptible to heat stress, especially under direct exposure to the sun’s rays since its evaporative cutaneous cooling mechanism is weak due to the presence of low density of sweat glands [4]. Heat stress causes summer anoestrous which hinders the reproductive efficiency in buffaloes [5]. The effect of heat stress is aggravated when heat stress is accompanied by high ambient humidity [6]. Buffaloes are considered as seasonal breeders, since most of the buffaloes come into oestrus during winter, and a very less number show oestrus in summer. Of late, it has been observed that heat stress can be more accurately quantified by temperature humidity index (THI) which takes into account the combined effect of air temperature and relative humidity (RH). When the mean THI was more than 80, there was a significant decrease in conception rate (CR) of lactating dairy cows [7]. Ravagnolo and Misztal [8] found a decrease in non-return rate (NR45) of 0.005 with per unit increase in THI above 68 on the day of service in Holstein cows. Variance of heat stress was found zero at THI 70 but it was started to increase with increase in THI and become equal to additive genetic variance at THI 84 for NR90 that indicated the presence of genetic variation in heat tolerance at high level of THI among Holstein cows. Phenotypic expression of any trait depends on both genotype and environment. Breeding value is twice the average deviation of its offspring from the population mean.

The estimation of expected breeding value for the traits defines the total genetic worth of the individual. The prediction of breeding values constitutes an integral part of most breeding programmes for genetic improvement of economic traits of Murrah buffaloes. The accuracy of estimating the breeding value of an animal is a major concern affecting the genetic progress due to selection [9]. The estimated breeding values for pregnancy rate (PR) of Angus heifers ranged from −0.02 to 0.05 [10].

The hypothesis of the study was to assess the role of environment, as well as genotype on the fertility traits of Murrah buffaloes. Until today, there is no report available on the assessment of EBV s for fertility traits of Murrah buffaloes under the non-heat stress, heat stress and critical heat stress zones (CHSZ) of a year. This investigation, therefore, is aimed to find out the expected breeding values (EBV) of fertility traits in three different defined zones of a year.

## Materials and Methods

### Ethical approval

This study is based on animal breeding data and it does not include animal experiment. So the ethical approval was not required.

### Herd location and general management

The present study was conducted on Murrah buffaloes maintained at Livestock Research Centre, National Dairy Research Institute (NDRI), Karnal, Haryana, India, located at 29° 42’ N latitude and 72° 02’ E longitudes in the bed of Indo-Gangetic alluvial plain. The climate of Karnal is subtropical in nature. There are four major seasons in the year *viz*. winter (December-March), summer (April-June), rainy (July-September) and autumn (October and November). The average temperatures in the four seasons were 15.6°C, 35.8°C, 28.7°C and 22.5°C and the average RH were 71.34%, 48%, 79% and 65%, respectively. Buffaloes were maintained in a loose housing system with brick on edges flooring under group management practice. The nutritional requirements of buffaloes were fulfilled through a standardized balanced ration of seasonal green fodders along with concentrates. Oestrus detection was carried out with the help of teaser Murrah bull (vasectomised bulls). Females detected in oestrus were inseminated with frozen semen of progeny tested bulls and pregnancy was confirmed after 45 days of insemination through rectal palpation as per standard management practices of the herd. Only healthy buffaloes were included while abortion, metritis, still birth, retained placenta and dystocia records were considered abnormal, and these were not included in the present study.

### Fertility data

The information related to fertility of Murrah buffaloes was collected from records maintained by dairy cattle breeding division, NDRI, Karnal. A total of 1379 records of 581 Murrah buffaloes under 1^st^, 2^nd^, 3^rd^ and 4^th^ parity spanned over a period of 20 years from July 1993 to December 2012 were collected. Three traits were generated such as service period (SP), PR and CR. The SP of buffaloes with more than 350 days was excluded from the study. The data were normalized with mean ± 3 standard deviation (SD) for SP. PR is a new and recent method of measuring fertility in buffaloes. It is defined as the percentage of non-pregnant buffaloes to become pregnant during each 21 days after voluntary waiting period (VWP)[11].

PR=21/(SP−VWP+11)

Where, VWP is the days in milk when buffaloes were not inseminated and VWP of Murrah Buffaloes has been standardized as 63 days at NDRI herd [12]. The CR of Murrah Buffaloes was computed with the following formula: CR=1/N * 100

Where, N=No. of inseminations required for pregnancy. A summary of the edited and normalized data sets is presented in Table-1.

**Table-1:** Edited and normalized data structure of fertility traits of Murrah buffaloes.

Parity	Initial observations	Observations under SP	Observations under PR	Observations under CR
1^st^	581	477	417	464
2^nd^	409	355	273	350
3^rd^	244	222	157	216
4^th^	145	138	108	135
Overall	1379	1192	955	1165

SP=Service period, PR=Pregnancy rate, CR=Conception rate

### Weather data and estimation of THI

Meteorological data pertaining to daily dry bulb temperature (T_db_) and RHwith the corresponding period of study were obtained from Central Soil and Salinity Research Institute, Karnal. Monthly average T_db_ (°C) and RH (%) were computed from daily weather information and subsequently the information were used to calculate the monthly average dew-point temperature (T_dp_) by the method given by Jensen *et al*. [13]. T_dp_ =116.9+237.3×ln(e)/16.78−ln(e), where, e (Kpa) = ambient vapour pressure.


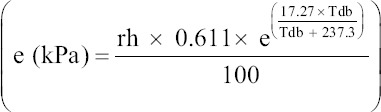


THI values were estimated on a monthly basis from January to December for each of 20 years (1993-2012) by using the formula given by Yousef [14].

THI=T_db_+(0.36×T_dp_)+41.2

### Statistical analysis

The data were distributed up to fourth parity in the present study. A total of 13 periods of calving were taken with an interval of 18 months artificial insemination cycle of a set of Murrah bulls for each period according to progeny testing programme under network project on Buffalo improvement. Ages of Murrah buffaloes at first calving were classified into t 3 Groups i.e., age Group 1: < 37 months; Age Group 2: 37-53 months and age group 3: > 53 months, using mean and one standard deviation in the population. The effect of non-genetic factors like parity, period of calving and age group at first calving on normalized traits viz; SP, PR and CR were assessed by using a fixed model least-squares analysis with least-squares maximum likelihood software (LSML) as suggested by Harvey [15]. The statistical model used was

Y_ijkl_=µ+pa_i_+p_j_+(AG)_k_+e_ijkl_

where, Y_ijkl_ is the observation on l^th^ Murrah buffalo belonging to k^th^ age group at first calving calved in j^th^ period of calving in i^th^ parity, and e_ijkl_ is random error ~NID (0, σ^2^_e_). The analysis of variance for the non-genetic factors affecting different fertility traits was computed and the difference of means between significant sub-classes was tested by using Duncan’s multiple range test (DMRT) as modified by Kramer [16]. The data were adjusted for the effects of significant non-genetic factors. Fertility traits like SP, PR and CR of Murrah buffaloes in each month of successful insemination were arranged along with the average THI of each month from January to December in each year and for 20 years, respectively.

### Threshold THI for fertility traits

The effect of THI on fertility traits of Murrah buffaloes was assessed by using a fixed Least-squares model with software LSML[15]. The generated THI values were classified into eight Groups such as 45.00-49.99, 50.00-54.99, 55.00-59.99, 60.00-64.99, 65.00-69.99, 70.00-74.99, 80.00-84.99 and 85.00-89.99, respectively.

The model used was

Y_ij_ = µ+THI_i_+e_ij_

Where, Y_ij_ is the observed fertility trait of j^th^ Murrah buffalo under i^th^ THI subclasses, µ=overall mean, THI_i_=fixed effect of i^th^ THI (1−8) and e_ij_ is random error ~NID (0, σ^2^_e_). Least-squares means along with standard errors for fertility traits under different THI subclasses were estimated. The significant effect of THI on fertility traits was assessed, and traits were adjusted with the significant effect of THI. The difference of means between THI sub-classes was tested using DMRT as modified by Kramer [16]. The threshold THI value for fertility traits was identified. Based on threshold THI value, two zones such as non-heat stress zone (NHSZ) and HSZ were determined in a year. Simple linear regression model was used to develop CHSZ within the HSZ. The regression model used was as follows.

Y_ij_=a+b x_i_+e_ij_

Where, a is intercept, b is regression coefficient and e_ij_ is random residual ~NID (0, σ^2^e). The zone where maximum increase in SP and decline in PR and CR were observed with per unit rise in monthly average THI was determined as the critical heat stress within the HSZ.

### Genetic evaluation of fertility traits in non-heat stress, heat stress and critical HSZs

Murrah buffaloes were genetically evaluated in NHSZ, HSZ and CHSZ based on their EBV for SP, PR and CR. Paternal half-sib correlation method [17] was used to estimate (h^2^) of different fertility traits. Similarly, (r) of each fertility trait was estimated by intra class correlation method [18]. The EBV for each fertility trait was estimated for buffaloes under each zone as follows.


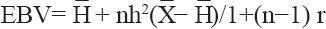


Where, 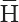
=Herd average of each fertility trait, n=number of records, h^2^=heritability estimate of fertility trait, r=repeatability estimate of fertility trait, 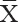
=average of each fertility trait.

## Results

**Figure-1:**
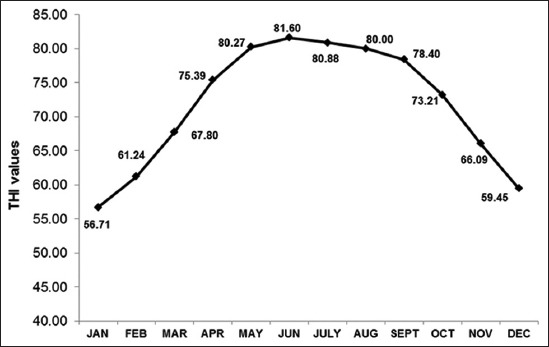
Monthly average temperature humidity index over 20 years (1993-2012) from January to December.

The month wise average dry bulb temperature varied from 12.43°C in the month of January to -32.54°C in may while RH ranged between 42.01% in April and 80.72% in August during the period. The lowest monthly average THI was 56.71 in January, and the highest mean THI was 81.60 in June over 20 years period as shown in Figure 1.

### Threshold THI for fertility traits

The parity (p<0.01) and period of calving (p<0.05) had significant effect on SP, but THI had highly significant effect (p<0.001) on all the fertility traits of Murrah buffaloes (Table-2) The least square means of SP, PR and CR are presented in Table-3. The overall least squares means for SP, PR and CR were 135.79 days, 0.38 and 68.80%, respectively. A distinct relationship was found between changes in fertility traits in relation to increasing in THI value. The average SP was estimated 127 days for THI subclass 70-74.99 that was increased to 162 days in THI subclass 75-79.99. The decrease in CR was evident from 76% in THI subclass 70-74.99 to 67% at THI value higher than 75. Similarly, PR was found declined from 0.41 to 0.25 in THI subclass 75-79.99 (Table-3). The negative effects of heat stress on fertility traits were observed above THI 75 which inferred that threshold THI value for SP, PR and CR were 75 in Murrah buffaloes.

**Table-2:** Analysis of variance (mean±SD values) of fertility traits in Murrah buffaloes.

Source of variation	SP (days)	PR	CR (%)
Parity	50654.78 (3)	0.27 (3)	366.89 (3)
Period of calving	15047.08 (12)	0.23 (12)	1373.90 (12)
Age group at first calving	378.11 (2)	0.18 (2)	200.37 (2)
THI	68265.77 (7)	1.08 (7)	3525.74 (7)
Error	7063.25 (1167)	0.15 (930)	920.05 (1140)

SP=Service period, PR=Pregnancy rate, CR=Conception rate, figures in parentheses indicate respective degree of freedom, p<0.05, p<0.01, p<0.001, THI=Temperature humidity index, SD=Standard deviation

**Table-3:** Least-squares means and standard errors of SP, PR and CR in Murrah buffaloes.

THI effects	SP (days)	PR	CR (%)
Overall (µ)	135.79±3.17 (1192)	0.38±0.02 (955)	68.80±01.18 (1165)
THI subclasses			
45.00-49.99	136.47^f^±17.31 (23)	0.31^c^±0.08 (20)	58.64^a^±06.42 (22)
50.00-54.99	130.67^d^±6.30 (166)	0.40^e^±0.03 (139)	66.67^c^±02.35 (164)
55.00-59.99	132.13^e^±7.20 (127)	0.44^g^±0.04 (105)	65.73^b^±02.70 (124)
60.00-64.99	116.55^b^±5.80 (196)	0.53^h^±0.03 (157)	76.87^h^±02.16 (194)
65.00-69.99	113.44^a^±10.66 (58)	0.39^d^±0.06 (44)	70.53^f^±03.95 (58)
70.00-74.99	127.17^c^±5.44 (223)	0.41^f^±0.03 (160)	75.92^g^±02.04 (217)
75.00-79.99	162.02^g^±7.09 (131)	0.25^a^±0.04 (107)	67.40^d^±02.68 (126)
80.00-84.99	167.84^h^±4.96 (268)	0.30^b^±0.03 (223)	68.63^e^±01.87 (260)

p<0.001; Means with dissimilar superscripts indicate a significant difference between THI sub-classes; Figures in parenthesis are a number of observations, THI=Temperature humidity index

In our study, monthly average THI value<75 were found for the months from October to March while mean monthly THI value>75 was observed during April to September over 20 years period. Accordingly, two zones in a year were identified such as NHSZ with average THI 56.71-73.21 and HSZ with average THI 75.39-81.60. May and June were thus identified as CHSZ with THI 80.27-81.60.

### Genetic evaluation of fertility traits under NHSZ, HSZ and CHSZ

The (h^2^) of SP, PR and CR was estimated as 0.06±0.03, 0.024±0.01 and 0.04±0.03, while the (r) estimates for the above traits were 0.13±0.04, 0.059±0.04 and 0.08±0.04, respectively (Table-4). Murrah buffaloes were genetically evaluated in relation to heat stress through assessing EB Vin each of the non-heat stress, heat stress and critical HSZ for all the fertility traits. The herd averages of SP, PR and CR over three different zones were estimated as 139 days, 0.36 and 68.92%, respectively. There was a trend in an increase in EBV of SP, decline in PR and CR from NHSZ to critical HSZ along with a decrease in the number of buffaloes (Table-5). The EBV of SP was estimated as 138.57, 139.62 and 140.92 days in three subsequent zones. The EBV of SP was found around 2 days higher under CHSZ as compared to NHSZ. The EBV of PR of Murrah Buffaloes was estimated as 0.362 under NHSZ that was declined to 0.358 under HSZ and further declined to 0.357 under critical HSZ. EBV of PR was decreased by 0.5% in CHSZ than NHSZ. The EBV of CR was 69.02%, 68.81% and 68.71% under NHSZ, HSZ and CHSZ, respectively (Table-5). The EBV of CR in Murrah buffaloes was found 0.31% lower in CHSZ as compared to NHSZ.

**Table-4:** Genetic parameters (heritability and repeatability estimates) of fertility traits of Murrah buffaloes.

Traits	Heritability (h^2^)	Repeatability (r)
SP (days)	0.06±0.03 (986)	0.13±0.04 (975)
PR	0.024±0.01 (782)	0.059±0.04 (755)
CR (%)	0.04±0.03 (964)	0.08±0.04 (957)

SP=Service period, PR=Pregnancy rate, CR=Conception rate, Figures in parenthesis are number of observations

**Table-5:** EBV for fertility traits of Murrah buffaloes in different zones.

Zones	Average THI value	EBV for service period (days)	EBV for pregnancy rate	EBV for conception rate (%)
NHSZ (October-March)	56.71-73.21	138.57 (427)	0.362 (396)	69.02 (415)
HSZ (April-September)	75.39-81.60	139.62 (313)	0.358 (261)	68.81 (311)
CHSZ (May, June)	80.27-81.60	140.92 (86)	0.357 (78)	68.71 (157)

NHSZ=Non heat stress zone, HSZ=Heat stress zone, CHSZ=Critical heat stress zone, Figures in parenthesis are a number of observations, EBV=Expected breeding values

## Discussion

There are several factors responsible for poor reproductive performances of animals. The climate data in terms of THI indicated that the reproductive performance of Murrah buffaloes was highly affected due to heat stress in the months with high THI values. Upadhyay *et al*. [19] earlier reported THI value 75 in the month of February and found to be increased to 85 in May and 95 in the month of July and August in Karnal. A comparable mean SP in Murrah buffaloes was estimated as 161.65 days [20]. Patil *et al*. [12] obtained a similar result of 0.36 Daughter PR in the 1^st^ parity after considering VWP 63 in Murrah buffaloes. The least square mean for CR was in confirmation of the findings of Nawale [21].

In this study, THI should be <75 for optimum reproductive performances of Murrah buffaloes and decline in fertility was observed with an increase in THI above 75. Ingraham *et al*. [22] indicated the decline in CR of Holstein cows from 55% to 10% with an increase in average THI of the day of service from 70 to 84. There is significant (p<0.05) decrease in the first service PR with increase in THI above 72 which corresponds to 25°C and RH 50% [23]. Hisashi *et al*. [7] identified the negative association of CR of lactating dairy cows with increase in THI in south western Japan at THI values higher than 80.

Our findings classified a whole year into three different zones *viz*.; NHSZ including months from October to March with mean THI 56.71-73.21, HSZ from April to September with mean THI 75.39-81.60 and CHSZ in the months of May and June with mean THI 80.27-81.60. The THI values are classified into three different classes of THI as comfortable (≤70), stressful (71-78) and extreme distress (>78)[24]. Armstrong [25] categorized THI values into five different classes as no stress with THI value <72, mild stress (72-78), moderate stress (79-88), severe stress (89-98) and dead cows with THI>98.

The (h^2^) and (r) estimates of SP in Murrah buffaloes were 0.06 and 0.13 in the present study. Thiruvenkadan *et al*. [26] reported a lower estimate of (h^2^) of SP in Murrah buffalo cows. The variation may be due to the difference in number of sires used for breeding, differences in management practices and environmental conditions such as ambient temperature, humidity and rainfall. Jamuna [27] obtained a comparable estimate for (r) of SP (0.15±0.05) in Murrah buffaloes. She has also estimated the (h^2^) and (r) of PR in Murrah buffaloes as 0.02 and 0.09±0.05, which are in agreement with our findings. Boichard and Manfredi [28] reported the (h^2^) and (r) estimates of CR of French Holstein dairy cattle as 0.02 and 0.032.

The EBV for SP was found increased from NHSZ to CHSZ, while the decline in EBV for PR and CR was observed from NHSZ to CHSZ in Murrah buffaloes. There was no report available regarding the estimation of breeding values for fertility traits of Murrah buffaloes in three different zones in a year under subtropical climate. The breeding value of CR and PR of Angus heifer cattle ranged between −0.2582 to 0.3401 and −0.4821 to 0.7793 [29]. Bermann *et al*. [10] also reported breeding value for PR of sires of Angus heifer cattle as −0.02-0.05.

## Conclusion

The result revealed that May and June months were the CHSZ for SP and PR, The months June, July and August months were the CHSZ for CR in Murrah buffaloes. The breeding value for fertility traits was influenced by THI. The EBV for fertility traits differed in three zones. In CHSZ the EBV of SP was found around 2 days higher than NHSZ. A decline of −0.5% in EBV of PR and −0.31% in the EBV of CR was observed in CHSZ as compared to NHSZ. As an overall conclusion, climatic factors seem to influence on fertility and in order to enhance the fertility performance of Murrah buffaloes proper management should be followed during the CHSZ.

## Authors’ Contributions

AKC designed the work. SD conducted study and analyzed the data. PRS and AU helped in the compilation of data. AKC and AS reviewed and edited the manuscript. VS and KMS helped in writing the manuscript. All authors read and approved the final manuscript.
